# Short and long-term carbon balance of bioenergy electricity production fueled by forest treatments

**DOI:** 10.1186/s13021-014-0006-1

**Published:** 2014-09-03

**Authors:** Katharine C Kelsey, Kallie L Barnes, Michael G Ryan, Jason C Neff

**Affiliations:** 1 Environmental Studies Program, University of Colorado, Boulder 80309, CO, USA; 2 Natural Resource Ecology Lab, Colorado State University, Fort Collins 80523, CO, USA; 3 Department of Geological Sciences, University of Colorado, Boulder 80309, CO, USA

**Keywords:** Forest carbon, Bioenergy, Climate change, Carbon emissions, Forest management, Fuels treatment, Wildfire, Forest Vegetation Simulator

## Abstract

**Background:**

Forests store large amounts of carbon in forest biomass, and this carbon can be released to the atmosphere following forest disturbance or management. In the western US, forest fuel reduction treatments designed to reduce the risk of high severity wildfire can change forest carbon balance by removing carbon in the form of biomass, and by altering future potential wildfire behavior in the treated stand. Forest treatment carbon balance is further affected by the fate of this biomass removed from the forest, and the occurrence and intensity of a future wildfire in this stand. In this study we investigate the carbon balance of a forest treatment with varying fates of harvested biomass, including use for bioenergy electricity production, and under varying scenarios of future disturbance and regeneration.

**Results:**

Bioenergy is a carbon intensive energy source; in our study we find that carbon emissions from bioenergy electricity production are nearly twice that of coal for the same amount of electricity. However, some emissions from bioenergy electricity production are offset by avoided fossil fuel electricity emissions. The carbon benefit achieved by using harvested biomass for bioenergy electricity production may be increased through avoided pyrogenic emissions if the forest treatment can effectively reduce severity.

**Conclusion:**

Forest treatments with the use of harvested biomass for electricity generation can reduce carbon emissions to the atmosphere by offsetting fossil fuel electricity generation emissions, and potentially by avoided pyrogenic emissions due to reduced intensity and severity of a future wildfire in the treated stand. However, changes in future wildfire and regeneration regimes may affect forest carbon balance and these climate-induced changes may influence forest carbon balance as much, or more, than bioenergy production.

## Background

Forests are an important component of the global carbon (C) cycle because of their role as a terrestrial C sink and their potential for long-term C storage. Many types of natural and human induced disturbances affect forest C storage including wildfire, insect outbreaks and drought. In the Intermountain West, forests are also commonly modified by fuel reduction treatments performed to reduce the risk of high severity wildfire, restore forests modified by fire suppression, and to protect homes in the wildland urban interface. Fuel reduction treatments also influence forest C balance both through their potential to modify fire behavior in recently treated forest stands, and because the treatments themselves remove woody biomass from the forest [1]-[4].

Forest fuel reduction treatments are designed to reduce fire severity by modifying surface fire behavior, reducing the risk of fire spreading from the ground surface to the forest canopy, and limiting fire spread within the forest canopy by decreasing canopy bulk density [5]. A number of studies indicate that fuel reduction treatments do reduce wildfire severity [6]-[10], and in some cases fuel treatments have been credited with altering the course of a wildfire when it encounters a previously treated area [11].

Forest fuel reduction treatments have also been proposed as a potential technique to limit C emissions from wildfire in some ecosystems [12],[13]. Forest treatments are designed to reduce mortality that would result from a high severity fire, and therefore they may ultimately limit wildfire C emissions to the atmosphere because C is maintained in the biomass of live trees [1],[3],[14],[15]. However, because forest treatments also remove woody biomass from the forest [2],[3], there is debate regarding whether the reduction in pyrogenic emissions is greater than the reduction in biomass during treatment [16]. Most pyrogenic emissions result from the combustion of surface fuels that burn comparably in both high and low intensity fires [17]. High intensity fires produce only 30% more direct emissions than low intensity fires, and fuel reduction treatments can remove as much or more biomass from the forest as is lost in a high intensity wildfire [16]. In some cases the total carbon emissions from treatment and subsequent wildfire may be greater in a treated forest stand than an untreated stand [18]. Furthermore, not all treated forest stands are likely to experience a wildfire because of the low probability of fire occurring in one location during a given time period [16], so some treated stands will have reduced C stores without any benefit from avoided pyrogenic emissions. Ultimately the C balance of a forest treatment will depend both on the fate of biomass harvested during treatment and the timeline of investigation.

**Figure 1:**
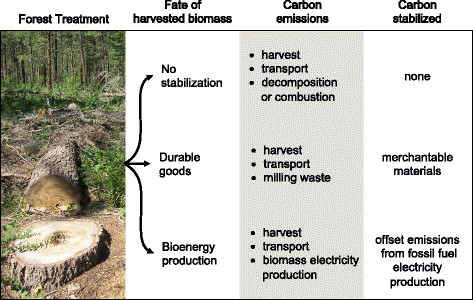
Sources of carbon emissions and types of carbon stabilization for different fates of harvested biomass following a forest fuel reduction treatment.

There are two potential fates of C in harvested biomass, emission to the atmosphere or stabilization, and the balance between emission and stability may shift depending on the timeline of interest (1, 10, 100 years). Immediately following a forest treatment, woody debris may be burned or left in the forest to decompose where it will result in emission of C to the atmosphere, or the C contained within harvested biomass may be may be stabilized if it used for timber and ultimately converted to durable goods [1]. An increasingly common fate for woody biomass is as a fuel for bioenergy-based electricity generation (Figure 1). The small diameter trees and understory biomass removed from forests during treatment can be directly combusted or converted to a synthetic natural gas, with both used for electricity production. Such use of biomass can stabilize C by offsetting C emissions from fossil fuels, and via sequestration of C during forest regrowth. However, bioenergy use also results in emissions of C during harvest, transport and electricity generation, with potential implications for overall C sequestration. Further, these processes can be of variable efficiency. For example, synthetic natural gas, or syngas, is produced from biomass by a thermochemical process called gasification (only partially efficient) that converts the biomass into fuel through partial oxidation at elevated temperatures [19]. High moisture content of the woody biomass can reduce the efficiency of the gasification process [20], producing further emissions. During electricity production, the syngas is combusted and the C within the syngas is emitted to the atmosphere.

**Figure 2:**
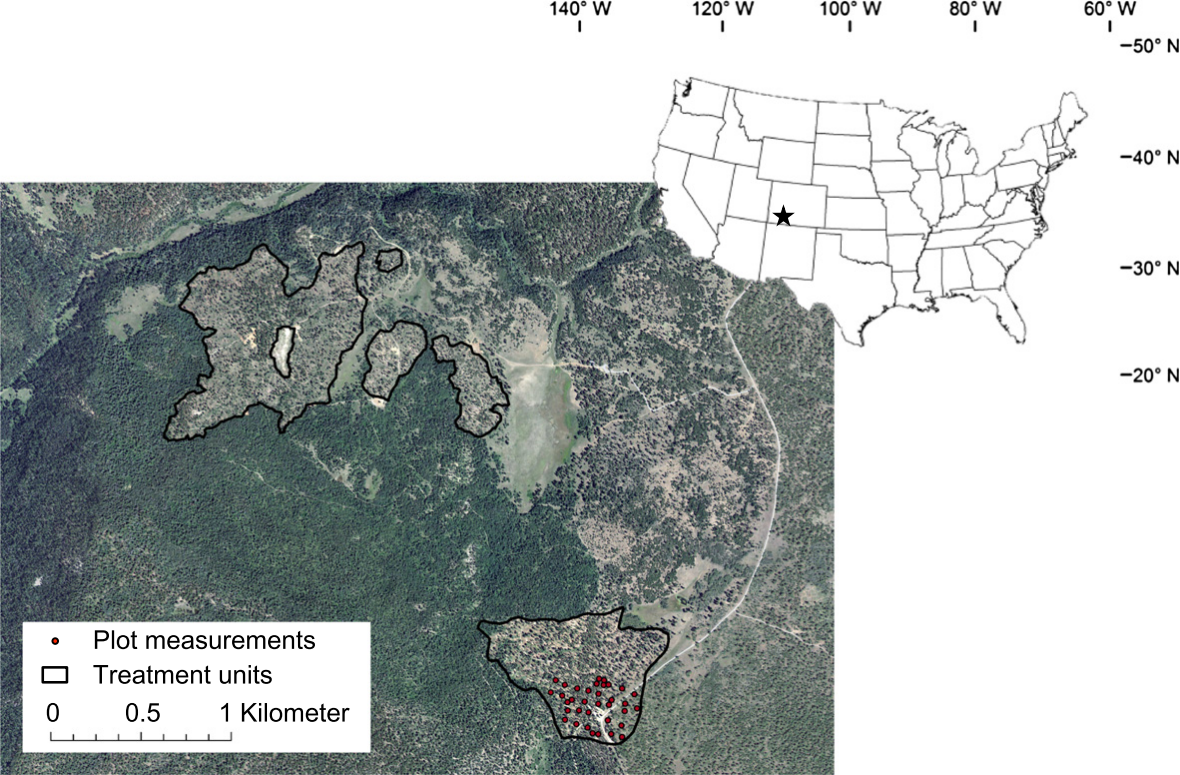
Arial image of study area within San Juan National Forest.

To investigate the C implications of fuel reduction treatments and the use of woody biomass for bioenergy electricity generation, we evaluate the C emissions and the short-term and long-term C balance of a 5 MW demonstration biomass gasification power plant in San Juan National Forest in southwest Colorado (Figure 2) under varying scenarios of forest treatment, disturbance and regeneration. We ask these questions:

(1) What are the relative C emissions of electricity generation from biomass and electricity generation from coal?

(2) How does the use of woody biomass for electricity generation change the C balance of forest fuel reduction treatments on a short-term (1 year) and long-term (100 years) time frame?

(3) How do treatment and bioenergy production affect forest C balance after a fire? How does post-fire C balance vary over differing scenarios of future fire intensity and regeneration?

## Results

### Relative carbon intensity of biomass and coal electricity production

**Figure 3:**
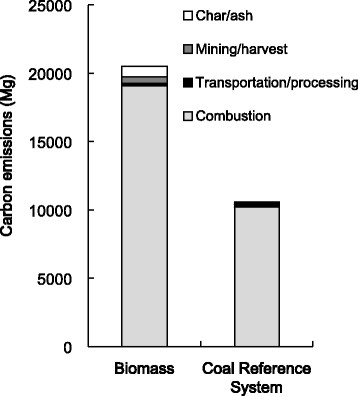
Carbon emissions for 8000 hours of 5 MW electricity produced from bioenergy and a coal reference system.

We found that electricity generation through biomass gasification produces almost twice the C emissions of a hypothetical coal reference system for the same amount of electricity production (Figure 3). The projected C emissions from biomass harvest, transportation, and electricity production for the amount of biomass necessary to fuel 5 MW electricity production for 8000 operational hours (estimated operation for one year), was 20,510 Mg C. The calculated emissions from the coal reference system for 8000 operational hours was 10,580 Mg C (Figure 3). These emissions are equivalent to emissions of 20.0 Mg C ha^−1^ for biomass electricity production and 10.3 Mg C ha^−1^ for coal electricity production according the number of hectares that must be treated annually on SJNF (1024 ha) to harvest the necessary amount of biomass.

### Short-term carbon balance of bioenergy production

The use of woody biomass for electricity generation reduces short-term net C emissions relative to other forest treatment scenarios investigated: one in which merchantable biomass is stored in durable goods, and a second ‘business-as-usual’ scenario in which the forest is treated, but all of the woody biomass removed from the forest is all allowed to decompose (Table 1). The bioenergy scenario reduces C emissions relative to the other two scenarios largely because the C emissions from bioenergy production (20,510 Mg C) were partially offset by the avoided emissions from coal-generated electricity production (10,580 Mg C). To determine emissions from the ‘durable goods’ scenario, we used simulations from our forest growth model that indicate that 49.5% of the biomass removed from the forest during treatment is considered merchantable. We assume that 60% of the total amount of C contained within the merchantable biomass (5611.6 Mg C) is sequestered in durable goods and therefore the total emissions from the forest treatment are equal to 14,082.4 Mg C or 13.8 Mg C ha^−1^. The net short-term emissions from bioenergy production were 9929 Mg C or 9.7 Mg C ha^−1^. The projected emissions for the no stabilization scenario are 19,694 Mg C, or 19.2 Mg C ha^−1^.

**Table 1:** Total carbon emissions, total carbon stabilized and net carbon emissions over one year from forest treatment considering three fates of harvested biomass: no biomass stabilized (business-as-usual scenario), merchantable timber stabilized in durable goods, and use of woody biomass in bioenergy production

	**Total emissions**	**C Stabilized in durable goods**	**C Offset through avoided coal emissions**	**Net emissions**
No stabilization (Business-as-usual)	−19.23	0.00	0.00	−19.23
C stabilized in durable goods	−13.75	5.48	0.00	−13.75
C stabilized in bioenergy production	−20.03	0.00	10.33	−9.70

### Long-term carbon balance of bioenergy production

**Figure 4:**
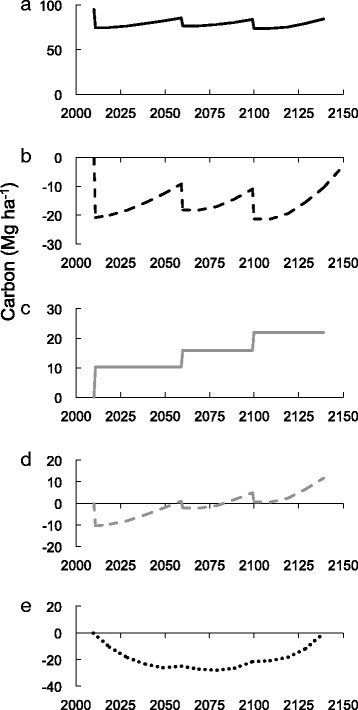
**Long-term carbon balance of a bioenergy electricity production.(a)** Total stand carbon **(b)** net stand carbon **(c)** cumulative carbon offset through bioenergy production and avoided coal emissions and **(d)** net stand carbon including carbon offset in a stand with repeated treatments. **(e)** Forest wide carbon balance with forest treatments to fuel bioenergy bioenergy production every year through 2100.

On a long-term time frame (>100 years), the use of woody biomass removed during forest treatments for electricity generation has a large effect on forest C balance. Repeated forest treatments reduce total stand C. Stand regrowth following treatment allows for some recovery of stand C storage through time (Figure 4a), but in many cases the repeated treatments necessary to maintain low risk of wildfire result in total stand C remaining below the pre-treatment stock (Figure 4b). Without any sequestration of the harvested biomass, the repeated treatments will result in a net emission of C to the atmosphere, even if there is forest regrowth between treatments. However, the use of woody biomass for bioenergy production sequesters C in the form of an offset of coal-generation C emissions. Through time as more treatments are completed, and more coal emissions are offset through bioenergy electricity production, the total amount of C sequestered increases (Figure 4c). We find that for the forest stand investigated here, the amount of C sequestered by bioenergy production via syngas and forest re-growth surpasses the C deficit incurred by the reduction in forest biomass before the end of this century (Figure 4d). If the treatments are repeated forest wide through 2100, the net C balance, including the coal offset, reaches zero around 2140 (Figure 4e). Following 2140, the net C balance of the forest is going to remain positive, even if the forest treatments are repeated indefinitely. The positive carbon balance is maintained because the amount of carbon offset due to bioenergy production increases with each treatment, even though repeated treatments continue reducing forest biomass below pre-treatment levels. In other words, once the cumulative amount of C removed during treatment is surpassed by the cumulative amount of C offset through bioenergy production and C sequestered during forest regrowth, the forest C balance will remain positive.

### Carbon balance of bioenergy treatments and future wildfire

**Figure 5:**
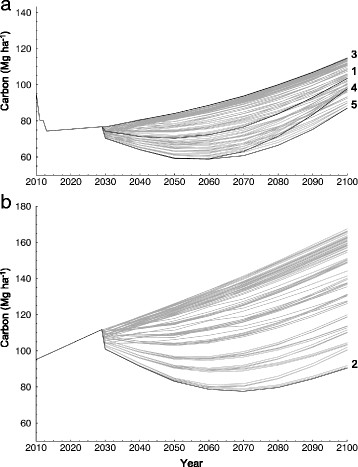
**Total carbon in a treated and untreated forest stand following wildfire.a)** Total stand carbon in a forest stand treated mechanically in 2011 and with prescribed fire in 2013, followed by multiple simulations of varying intensity wildfire and regeneration in 2030; **b)** total stand carbon in an untreated forest stand with simulations of varying intensity wildfire and regeneration in 2030. Dark lines (numbered 1, 2, 3, 4, 5) represent the selected comparisons presented in Table 1.

Simulated post-wildfire stand C stocks vary depending on wildfire intensity and stand treatment history. We found that the range of simulated post-wildfire total stand C values was greater in an untreated stand than in a treated stand. Potential total stand C in 2100 ranged from 87 to 166 Mg C ha^−1^ in the untreated forest, and from 75 to 109 Mg C ha^−1^ in the treated forest (Figure 5). The untreated forest also had a greater minimum and maximum total stand C value than the treated forest. However, the introduction of fuel treatments modified fire behavior; we find that in a selected comparison of a treated and untreated forest stand that both burn in a wildfire, the treated stand maintains more live tree C and total stand C in 2100 than the untreated stand, due to the difference in wildfire fire intensity and severity following forest treatment (Table 2). In this example, the treatment results in 14.76 Mg ha^−1^ of avoided pyrogenic C emissions, plus 10.3 Mg ha^−1^ of C due to the offset of C emissions from the replacement of coal energy production with bioenergy production.

**Table 2:** Effects of treatment on fire behavior and forest carbon balance

					**2100**		
**Plot**	**Treated**	**Bioenergy**	**Wildfire intensity**	**Regeneration**	**Δ Live Tree C (Mg ha**^**−1**^**)**	**Δ Total Stand C (Mg ha**^**−1**^**)**	**Net C balance (Mg ha**^**−1**^**)**
1^a^	Y	Y	Moderate	Normal	35.98	10.01	20.31
2^b^	N	N	High	Reduced	−31.19	−4.75	−4.75

^a^Wildfire parameters in FVS: windspeed, 16.09 km/hr; fuel moisture, very dry; 40% stand burned; season, ‘before fall’; regeneration, 300 trees per acre.

^b^Wildfire parameters in FVS: windspeed, 64.37 km/hr; fuel moisture, very dry; 90% stand burned; season, ‘before fall’; regeneration, 0 trees per acre.

A selected comparison of the effects of forest treatment on year 2100 forest carbon balance following wildfire. The effects of the treatment and subsequent changes in wildfire intensity and regeneration are reflected in the ‘ Δ Live Tree C’ and ‘Δ Total Stand C’ columns, and the effect of treatment and bioenergy production is shown in the ‘Net C balance’ column. ‘Wildfire Intensity’ is a qualitative descriptor of the wildfire parameterizations used in the forest growth model. The Plot column indicates the line number depicted in Figure 5.

The forest treatment and bioenergy production also have the potential to reduce C emissions even without avoided pyrogenic emissions due to the offset of coal-generated emissions. In a selected comparison of two treated forest stands with low severity fire, the use of biomass for electricity production increased the net C balance from 19.50 Mg ha^−1^ in a treatment without bioenergy production to 29.80 Mg ha^−1^ in a treatment with bioenergy production (Table 3). Finally, regeneration following wildfire also influences forest net C balance in 2100. In a comparison investigated here, a forest stand with normal regeneration following a high severity fire reaches a positive C balance by 2100, whereas a stand that burns in the same wildfire but does not regenerate has a negative C balance in 2100 (Table 4).

**Table 3:** Effects of bioenergy production on forest carbon balance

					**2100**		
**Plot**	**Treated**	**Bioenergy**	**Wildfire intensity**	**Regeneration**	**Δ Live Tree C (Mg ha**^**−1**^**)**	**Δ Total Stand C (Mg ha**^**−1**^**)**	**Net C balance (Mg ha**^**−1**^**)**
3^a^	Y	Y	Low	Normal	19.53	19.50	29.80
3^a^	Y	N	Low	Normal	19.53	19.50	19.50

^a^Wildfire parameters in FVS: windspeed, 16.09 km/hr; fuel moisture, moist; 40% stand burned; season, ‘early season’; regeneration, 300 trees per acre.

A selected comparison of the effects of bioenergy production from biomass harvested during forest treatment on year 2100 forest carbon balance following wildfire. The effects of bioenergy production on carbon balance are evident in ‘Net C balance’ column, and would be the same regardless of wildfire intensity or regeneration parameters. ‘Wildfire Intensity’ is a qualitative descriptor of the wildfire parameterizations used in the forest growth model. The Plot column indicates the line number depicted in Figure 5.

**Table 4:** Effects of regeneration on forest carbon balance

					**2100**		
**Plot**	**Treated**	**Bioenergy**	**Wildfire intensity**	**Regeneration**	**Δ Live Tree C (Mg ha**^**−1**^**)**	**Δ Total Stand C (Mg ha**^**−1**^**)**	**Net C balance (Mg ha**^**−1**^**)**
4^a^	Y	Y	High	Normal	−17.68	2.65	12.95
5^b^	Y	N	High	Reduced	−27.06	−8.15	−8.15

^a^Wildfire parameters in FVS: windspeed, 64.37 km/hr; fuel moisture, very dry; 90% stand burned; season, ‘before fall’; regeneration, 300 trees per acre.

^b^Wildfire parameters in FVS: windspeed, 64.37 km/hr; fuel moisture, very dry; 90% stand burned; season, ‘before fall’; regeneration, 0 trees per acre.

A selected comparison of the effects of varying regeneration and bioenergy production on year 2100 forest carbon balance following wildfire. The effect of regeneration on carbon balance is shown in the difference in ‘Δ Total Stand C’ and the effects of bioenergy production plus regeneration is shown in the ‘Net C balance’ column. ‘Wildfire Intensity’ is a qualitative descriptor of the wildfire parameterizations used in the forest growth model. The Plot column indicates the line number depicted in Figure 5.

## Discussion

Here we explore the short-term (~1 year) and long-term (~100 year) C balance of a demonstration fuel reduction treatment with use of woody biomass for bioenergy electricity production. We find that although bioenergy is a more C intensive energy source than coal, the use of bioenergy production in this forest reduces overall treatment emissions relative to other treatment scenarios investigated. We also find that while repeated forest treatments can lower forest C storage, when the harvested biomass is used for electricity generation, the C sequestered by offsetting coal-generated C emissions results in a net C sink by 2140. In addition to the C benefit obtained through bioenergy production, the forest treatment may also reduce C emissions through avoided pyrogenic emissions in a subsequent wildfire. However, the C benefit incurred through the bioenergy production is comparable, or in some cases smaller, than the changes in stand C due to variable wildfire intensity or regeneration. Future changes in disturbance or regeneration regimes also have the potential to affect forest C balance in addition to the use of bioenergy for electricity generation.

### Short-term carbon balance of bioenergy production

Bioenergy electricity generation results in lower C emissions to the atmosphere than the other treatment scenarios investigated (Table 1). There are several factors that explain the relatively low emission from the bioenergy scenario. First, although biomass electricity generation produces C emissions through the combustion of biofuel, nearly half of the C emissions from bioenergy production are offset by avoided coal emissions. Secondly, there are relatively few C emissions from biomass waste in the bioenergy production process because the bioenergy production facility used as a reference for these calculations has few limitations regarding the size of woody material that can be used in electricity production. Therefore, small biomass scraps that cannot be used in durable goods can be utilized in bioenergy production. Furthermore, the reduction in C emissions of the bioenergy scenario relative to the durable goods scenario may be even greater than that represented here. Transportation emissions from the treatment site to a mill were assumed to be the same as the emissions recorded for transporting the biomass from the treatment site to the bioenergy production site, even through there is currently no mill located within that proximity to the forest. Finally, the durable goods scenario does not include any further emissions incurred for transportation of the final product, or during processing. However, emissions incurred during processing, such as from milling waste, can be difficult to estimate because many mills will use waste to generate electricity or another type of non-durable product [21].

The relative future emissions of the these three scenarios will vary depending on several factors including the distance between the harvest site and the bioenergy facility, the efficiency of the bioenergy production process, and the size of biomass available for harvest. The distance between the harvest site and the bioenergy production facility can impact the carbon balance of the bioenergy production process because a longer haul distance that requires greater C emissions during transportation of biomass may reverse the carbon benefit provided from bioenergy production. We calculated two maximum haul distances for this study: the maximum haul distance at which bioenergy production will provide a carbon benefit over the durable goods scenario, and the maximum haul distance at which the bioenergy scenario will provide a carbon benefit over the no stabilization scenario. In both cases, the maximum haul distance is great enough that biomass could be retrieved from all available regions of San Juan National Forest. In addition to changes in the haul distance of harvested biomass, there also may be future changes in the efficiency of the bioenergy production process, which would increase the carbon benefit provided by this scenario relative to the other two. Finally, the size of biomass available for harvest will vary with time and in the future there may be fewer trees of merchantable size, which would decrease the amount of biomass that could be stored in durable goods.

### Long-term carbon balance of bioenergy production

Treatments designed to reduce the risk of high intensity wildfire necessarily lower the amount of forest biomass present on the landscape because biomass is removed from the forest [1]-[4]. When these treatments are periodically repeated in order to maintain reduced fire risk, they result in lower C storage on the landscape (Figure 4a). If the treatment reduces emissions from a future wildfire by an amount greater than the amount of C removed during treatment, then the treatment will result in a net C benefit. However, this is only possible in the case of a future wildfire, and considering only a portion of the landscape is burned in a wildfire each year, many treated areas will not be subsequently burned during the lifespan of treatment effectiveness [16].

We find that in the bioenergy scenario we investigate here, repeated treatments with bioenergy electricity production result in a net C benefit even without a future wildfire. Because some C is ‘sequestered’ from every treatment through the offset of coal energy production (Figure 4c), and C is also taken up through forest regrowth, the cumulative C emission to the atmosphere is reduced with every treatment. In the case of the forest stand investigated here, repeated treatments result in a net C balance of zero by 2080 (Figure 4d). A forest level analysis, assuming continued operation of the bioenergy plant every year through the end of the century indicates that the net C balance of the forest reaches 0 by the year 2140, and will remain positive thereafter (Figure 4e). The results we report here are contingent on the size of the forest investigated and the use of coal as the energy reference system, however these results are highly applicable to decision makers in Southwest Colorado. Because current forest policy mandates forest treatments to reduce wildfire risk [22], these types of forest treatments are routinely performed on Western forests whether or not there is an opportunity to produce electricity from the harvested biomass. Our results indicate that the use of biomass for electricity generation may reduce the overall C emissions resulting from these ongoing forest treatment practices.

### Carbon balance of bioenergy treatments and future wildfire

**Figure 6:**
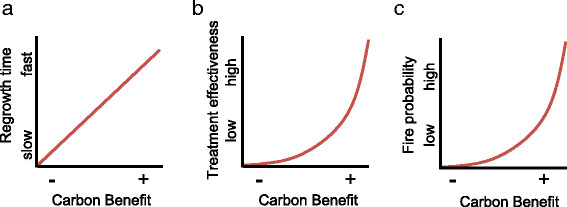
**Conceptual model of the effect of (a) regrowth time, (b) treatment effectiveness, and (c) fire probability on carbon benefit of forest treatment.** The timeframe is assumed to be smaller than the disturbance cycle in the specified forest.

In addition to the C offset from bioenergy production, forest treatments may also provide a C benefit by reducing emissions from a future wildfire. We find that in a comparison of two scenarios of future wildfires occurring in treated and untreated stands, the treated stand provides a C benefit due to avoided emissions as a result of the treatment. However, over larger temporal and spatial scales, the C benefit of forest treatment is contingent on three factors: (1) the rate of forest growth following treatment, (2) the effectiveness of the treatment in modifying fire behavior, and (3) the probability of future wildfire (Figure 6).

#### Forest growth

The C benefit of forest treatments is dependent on the rate of forest growth following treatment (Figure 6a). Indeed the long-term C balance of all forest disturbances is dependent on the forest recovery and the frequency of the disturbance. Over a time scale of one hundred to several hundred years, forest disturbances including treatment or wildfire will only result in forest C loss if the forests are not allowed to recover in the time period between disturbances [16],[23]. In other words, net C loss to the atmosphere occurs in instances where the disturbance interval is shorter than the time required for the forest to regrow to its pre-disturbance state, or where the forest experiences a permanent conversion to a different vegetation type. We find that in the scenario we investigate here, repeated treatments necessary to maintain the forest at a low risk of high severity wildfire do not allow the forest to recover to its pretreatment C stock (Figure 4b). However, because bioenergy production offsets some C emissions with every treatment, the net C balance of these treatments in our demonstration stand eventually does reach zero around the end of the century (Figure 4d), and forest wide net C balance equals zero by 2140.

#### Treatment effectiveness

The effectiveness of forest treatments is critical in determining the ultimate C benefit of a forest treatment; a treatment that does not effectively reduce future emissions will incur a low, or no, C benefit, whereas a treatment that is highly effective in decreasing future wildfire emissions will incur a larger C benefit. Ultimately this effect will saturate when so much biomass has been removed from the forest that further treatment will not further reduce fire potential (Figure 6b).

The effects of forest treatments on wildfire behavior are difficult to characterize, but there are many studies indicating that treatments can effectively reduce fire behavior and post-fire mortality in dry western forests. A comparison of fire severity indices, fireline intensity, stand characteristics and post-fire recovery in treated and untreated stands in New Mexico and Arizona indicates that fire severity was lower in treated areas, and more aggressive treatments made stands less susceptible to crown fire [6]. Analyses with satellite data indicate that treatments reduced wildfire severity and also changed the progress of the Rodeo and Chediski fires in Arizona [11], and Pollet and Omi [7] found that among four sites in the Western US revisited following wildfire, crown fire severity was mitigated (fire severity and crown scorch was lower) in stands that had some type of fuel reduction treatment. Investigations of stand structure, composition and mortality following a wildfire in adjacent treated and untreated stands indicate that treated stands have lower post-wildfire mortality [24], and greater C storage in live tree C pools [18],[25].

Given the current understanding of the effects of forest treatments on wildfire behavior, it is nearly impossible to definitively determine how treatments will affect wildfire C emissions. However, projections of avoided pyrogenic emission due to forest treatments are critical in determining future forest C balance. In the selected scenario we investigate here, the forest treatment results in 14.76 Mg ha^−1^ of avoided C emissions, while the C emissions offset through bioenergy electricity production is 10.3 Mg ha^−1^, indicating that a reduction from high intensity to low intensity fire in this region could potentially have a larger effect on stand C balance that the use of bioenergy along. Projections of pyrogenic C emissions and the effects of forest treatment on fire behavior can have substantial implications for projecting future forest C balance and therefore this is an area of research that deserves careful analysis in the future.

#### Wildfire probability

In addition to forest growth and treatment effectiveness, the C benefit of a forest treatment is also dependent on the probability of a future wildfire in the treated area (Figure 6c). If the area of the treatment does not experience a wildfire during the lifespan of the treatment effectiveness, then the emissions resulting from the treatment are not offset by a subsequent reduction in pyrogenic emissions. Within San Juan National Forest there are 171,400 hectares of ponderosa pine forest, and according to data from LANDFIRE, 56,600 hectares of ponderosa pine on SJNF burned between 1999 and 2010, indicating that an average of 0.3%, or 5150 hectares, of ponderosa pine forest on SJNF burned each year during that time period. Given the relatively small probability of a specific treated stand experiencing a fire during the life span of the treatment, and the fact that 1024 ha must be treated annually to harvest enough biomass to fuel the plant, the primary C benefit of the treatment will likely come from the bioenergy production and the associated C offset, not avoided pyrogenic emissions.

In the future, wildfire probability may be influenced by climate-induced changes in wildfire regimes. Recent analyses indicate that annual wildfire area burned is correlated with climate [26],[27]; large wildfire activity and wildfire season duration have increased since the mid-1980’s [28] and in many western states the annual wildfire area burned may double by the end of the century [27]. An increased probability of wildfire also increases the potential for a stand treatment to incur a C benefit through avoided pyrogenic emissions. Under future conditions of more frequent wildfire in this region, the potential for stand treatments to provide a C benefit may increase, although this increase will likely remain small, as the probability of wildfire in one particular location is low.

In addition to wildfire size and frequency, potential future changes in wildfire intensity and severity may also have consequences for ecosystem recovery. In a study surveying 10 sites following stand-replacing wildfire in ponderosa pine ecosystems, Savage and Mast [29] found that only 50% of the sites experienced any regeneration, and the remaining sites appeared to have transitioned to grassland or shrub land with reduced potential to recover C. In our analyses we find that in the absence of regeneration following wildfire, it takes longer for the forest to re-sequester C emitted during the wildfire. Indeed, the difference in year 2100 total stand C stocks between a burned stand with reduced regeneration and a burned stand with normal regeneration is as large as the C offset obtained through bioenergy production (Tables 3 and 4). While more frequent wildfires in the future may mean that forest treatments have a greater potential to reduce total C emissions, reduced forest regeneration or recovery may lessen the potential C benefit of forest treatment for bioenergy. Further investigation of treatment effects on fire behavior and projected trends in forest regeneration and recovery following disturbance are critical in determining the ultimate C balance of treatments and bioenergy production.

## Conclusion

Forest treatments influence forest C balance by removing woody biomass from the forest, and also by affecting future wildfire behavior. We find that the use of harvested biomass for electricity generation can reduce C emissions to the atmosphere by offsetting emissions from fossil fuel electricity generation, and potentially avoiding pyrogenic emissions by reducing the intensity of a future wildfire. However, future variations in fire frequency and intensity, and in forest regeneration following disturbance, may also influence forest C stocks and in some cases these changes in forest C stocks are larger than the C sequestered through offsetting coal emissions.

## Methods

### Study site

The site of this study was the Turkey Springs Demonstration Area in the eastern portion of San Juan National Forest (SJNF). The site is located at 37° 15’ N and 107° 10’ W, and at 2500 meters elevation (Figure 1). Average maximum and minimum temperatures are 14.2°C and −2.16°C respectively, and average annual precipitation is 618.4 mm (http://prismmap.nacse.org/nn/). The total area of the Turkey Springs demonstration site is 116 hectares. The site is broken into five units, Units 1–5. All biomass measurements were made on Unit 5, which is 39 hectares in size. Biomass values from Unit 5 were used for the area of all units, which are covered by the same vegetation type. Units 1 through 4 have a similar management history as Unit 5; records of historical treatment activities maintained by the Forest Service indicate that all units were harvested by individual tree selection in 1967, parts of Units 3, 4 and 5 were commercially thinned in 1968, and the east half of Unit 5 was logged again in 1983. Vegetation present at the site is dominantly ponderosa pine (*Pinus ponderosa* Dougl. Ex Laws) with scattered pockets quaking aspen (*Populus tremuloides* (Michx.)), douglas-fir (*Pseudotsuga menziesii* Mirb.), white fir (*Abies concolor* (Gord. & Glend.)) and gambel oak (*Quercus gambelii* Nutt.).

### Forest biomass

Forest biomass present before treatment was measured in 2011 using 34 circular inventory plots, each 80 m^2^ in area (diameter = 10.24 m). Within each plot the diameter of every tree over 1.37 m tall was measured at 1.37 m to obtain a measure of diameter at breast height (DBH) for all trees within the plot. Aboveground live tree biomass was calculated from tree DBH using allometric equations from Jenkins et al. [30] and Kaye et al. [31]. Aboveground live tree biomass for the plot was determined as the sum of all trees present on that plot, and C was calculated as 50% of dry biomass [32]. Biomass inventory plots were re-measured in 2012 following the fuel reduction treatment using the same inventory methodology. The amount of forest biomass removed during the demonstration fuel reduction treatment was determined by weighing all woody material as it was removed from site. Dry biomass was determined by assuming 45% moisture content of material removed.

### Bioenergy and coal reference systems

The reference bioenergy system we investigate is assumed to produce 5.0 Megawatts of electrical power (MW) and operate 8000 hours per year (91% operating time). 58,400 Mt of wet biomass (45% moisture) will be necessary to fuel 5 MWe production. Once the raw wood is harvested and transported, it is dried to 20% moisture. The wood is then converted to syngas, which is used to fuel the internal combustion engines of the plant.

Total C emissions from a hypothetical 5 MW coal reference system were calculated to compare the C intensity, or the C emissions per unit energy, of the coal to that of the bioenergy electricity generation system. Coal was chosen as a reference system because coal is the primary energy source for Southwest Colorado [33]. Total emissions from the coal reference system included three components: mining, transportation, and combustion. Mining, transportation and combustion emissions were calculated based on values from Kerr, Mann and Spath [34]. The coal reference system was assumed to operate at 32% efficiency and use coal with 70% C content [34]. We also calculated ash production during coal combustion, and subtracted ash C content from total C emissions from the system. No assumption was made regarding the eventual fate of the ash; for this work here we do not consider further emissions from ash decomposition.

### Bioenergy and forest harvest emissions

Expected C emissions from the bioenergy power plant are derived from three primary sources: emissions associated with biomass removal and transport, emissions from syngas production, and emissions from syngas combustion. The first source of emissions from bioenergy production was emissions associated with biomass harvest and transport. Operational hours for each piece of equipment used in all 5 Units (116 hectares) of the fuel treatment area were tracked by the biomass harvesting team and used to calculate total emissions [35]. Emissions from transportation of biomass from the treatment site to the bioenergy facility were also calculated based on the total hours of operation, and the average fuel consumption per hour for both gasoline and diesel [35].

We calculated C emissions for syngas production (gasification) based on projections that 8000 operational hours are necessary to produce 5 MW of electricity, and C emissions from biomass gasification obtained from Basu [2010] [36]. Total emissions from combustion within the bioenergy system included both syngas production for electricity generation, and also the combustion of natural gas necessary to maintain the high internal temperature of the gasification operation. Using the projected syngas composition and the volumes of biomass and natural gas necessary for 5 MW of electricity production, gas volumes for each constituent of gas were converted to grams of C, and summed to determine total projected emissions for syngas combustion. C emissions from natural gas combustion were determined from projections of net gas consumption and gas composition obtained Lieuwen et al. [37]. All char produced through the gasification process was considered an emission to the atmosphere.

### Forest growth and disturbance modeling

Forest growth and the effects of future disturbance were modeled using the Central Rockies variant of the Forest Vegetation Simulator (FVS) and the Fire and Fuels Extension (FEE) [38]. FVS is a widely used forest growth and yield model, and is frequently used to inform ponderosa pine management [39]. The FEE can be used to predict tree mortality, fuel consumption and C emissions following fire based on inputs of weather, fuel, and stand characteristics [40]. We used FVS-FEE to simulate the C emissions associated with the fuel reduction treatment in 2011 and the prescribed fire following treatment in 2013. FVS was also used to determine live tree and total stand C following repeated treatments recurring every 40 years designed to reduce stand basal area to 7.4 m^2^ (80 ft^2^).

We also investigated three scenarios concerning the fate of biomass removed during forest treatment: a ‘no stabilization’ scenario which is considered the ‘business-as-usual scenario, a scenario where biomass is used for durable goods, and finally one in which all biomass removed during treatment is used for bioenergy electricity production. FVS was used to simulate the amount of biomass removed from the forest during treatment. Projections from FVS were also used to determine what fraction of the biomass removed was considered merchantable, defined as a bole with a top diameter greater than 10.2 cm. Forty percent of merchantable material was assumed to be lost as milling waste, and the remaining 60% converted to durable goods [2],[21].

FVS was also used to project changes in total stand C and live tree C associated with a future wildfire at this site in 2030. Wildfires tend to burn in a highly heterogeneous manner, with patches of lightly burned and intensely burned forest depending on variables including weather conditions and landscape patterns [41]. Because it is impossible to know the severity and intensity of a future wildfire at this site, we simulated 96 future wildfires by varying the wildfire controls present within FVS. The wildfire controls present within FVS are: wind speed, fuel moisture, air temperature, percentage of stand burned, and the season of the fire. For our model simulations, we varied wind speed between 16.09, 32.18, 48.28 and 64.37 kilometers per hour, fuel moisture was varied between ‘very dry’ and ‘moist’ settings, percentage of stand burned was varied between 40 and 90 percent in increments of 10, and the season of the fire was set as ‘early season (compact leaves)’ and ‘after greenup (before fall)’. Air temperature was maintained at 29.4 degrees Celcius. We also varied the prescription of future forest regeneration to account for uncertainties in regeneration under future climate and wildfire conditions. Regeneration scenarios were based on empirical data from Savage and Mast [29], indicating that 50% of ponderosa pine sites investigated following a stand-replacing fire did not regenerate. No regeneration was prescribed in either the treated or untreated stand until after the wildfire. All fire and regeneration scenarios were run on a treated and untreated forest stand for a total of 384 simulations.

## Abbreviation

C: Carbon

## Competing interests

The authors declare that they have no competing interests.

## Authors’ contributions

KCK conducted all simulation modeling, and did most of the writing of the manuscript. KLB designed and led field data collection, and performed calculations of emissions from bioenergy production. MGR designed conceptual model of forest treatment affects on C benefit and assisted with writing and editing the manuscript. JCN developed the study idea and assisted with writing and editing the manuscript. All authors read and approved the final manuscript.
